# Clinical characteristics, fracture patterns, and neurological outcomes in spinal cord injury patients: A prospective cohort study from a high-volume trauma centre

**DOI:** 10.1097/MD.0000000000046723

**Published:** 2025-12-19

**Authors:** Zia Ur Rehman, Syed Shayan Shah, Muhammad Sohaib Khan, Bilal Khan, Syed Jawad Ahmad, Muhammad Aamir

**Affiliations:** a Department of Neurosurgery, Lady Reading Hospital, Peshawar, Khyber Pakhtunkhwa, Pakistan; b Department of Neurosurgery, Lady Reading Hospital, Peshawar, Khyber Pakhtunkhwa, Pakistan; c Department of Neurosurgery, Lady Reading Hospital, Peshawar, Khyber Pakhtunkhwa, Pakistan; d Department of Neurosurgery, Lady Reading hospital, Peshawar, Khyber Pakhtunkhwa, Pakistan; e Department of Neurosurgery, Lady Reading Hospital, Peshawar, Khyber Pakhtunkhwa, Pakistan; f Department of Neurosurgery, Lady Reading Hospital, Peshawar, Khyber Pakhtunkhwa, Pakistan.

**Keywords:** ASIA grade, conservative versus surgical management, fracture type, functional outcome, neurological improvement, spinal fracture

## Abstract

Spinal fractures are a major cause of disability, with outcomes influenced by fracture type, neurological status, and treatment approach. Identifying predictors of neurological improvement is essential for optimizing patient management. To assess the association between fracture type and clinical outcomes and to determine factors linked to neurological improvement in patients with spinal trauma. This prospective cohort study included adults (≥18 years) of either gender with traumatic spinal injuries resulting from road traffic accidents (RTAs), diving, or falls, admitted to a tertiary trauma center. Outcomes included neurological status assessed by the American Spinal Injury Association (ASIA) Impairment Scale at admission, discharge, and three-month follow-up; ambulatory status; pain via visual analogue scale (VAS); and neurological improvement (change in ASIA grade). Data on fracture type, mechanism of injury, treatment modality, physiotherapy, and in-hospital complications were recorded. Associations were analyzed using chi-square tests, with *P* < .05 considered significant. RTAs were the most common mechanism (49.6%). The mean age was 32.2 ± 11.4 years, and 65.9% were male. Fracture type was significantly associated with ambulatory status (*P* < .001; 96.8% ambulatory for wedge vs 41.4% for burst fractures), follow-up ASIA grade (*P* < .001), VAS score (*P* = .001), and treatment modality (*P* < .001). Wedge fractures and spinal cord injury without radiologic abnormality (SCIWORA) were linked to better outcomes, whereas burst fractures showed higher neurological deficits (93.5% normal for wedge vs 12.9% for burst). In the follow-up subgroup, neurological improvement correlated significantly with fracture type (*P* < .001), treatment modality (*P* < .001), initial ASIA grade (*P* < .001; OR 15.2 for grade D vs A), physiotherapy (*P* = .047), follow-up VAS score (*P* = .006), and complications (*P* = .047). Greater recovery occurred in patients managed conservatively and those with initial ASIA grades C and D. Fracture type, treatment approach, and rehabilitation significantly influence neurological outcomes in spinal trauma. Wedge fractures and SCIWORA demonstrated favorable prognoses, while early physiotherapy and conservative management promoted recovery. These findings can guide clinicians in treatment selection, counseling, and rehabilitation to enhance neurological and functional outcomes.

## 1. Introduction

Spinal injuries are a significant source of morbidity, especially in young and otherwise healthy individuals, frequently occurring from high-energy trauma such as road traffic accidents (RTAs), diving into shallow water, and falls from height. RTAs often result in injuries to the cervical and thoracolumbar spine due to causes including direct impact, abrupt deceleration, and vigorous twisting or bending of the spine.^[1–3]^ Diving, particularly head-first entrance into shallow water, often results in cervical spine injuries that can have severe neurological repercussions.^[1]^ Falls, especially from considerable heights, are a primary cause of thoracolumbar fractures.^[1,4]^

The occurrence of spinal cord injuries (SCIs) resulting from diving or falls into shallow water varies between 1.2% and 21%, primarily impacting the young males. Worldwide, RTAs and falls constitute the predominant causes of spinal injuries, with RTAs being marginally more common. Thoracolumbar trauma is recorded more commonly than cervical spine injury among the affected vertebral areas.^[4,5]^ The categorization of fractures is essential for treatment planning and prognosis. The Arbeitsgemeinschaft für Osteosynthesefragen classification system is extensively utilized to classify spinal fractures into Type A (compression), Type B (disruption of the posterior ligamentous complex), and Type C (translation or rotational injuries).^[6]^ A local investigation indicated that Type A fractures were the most prevalent (43.3%), followed by Type B fractures (37%), with neurological impairments noted in more than half (54.3%) of the patients.^[5]^

SCI present with a broad spectrum of neurological outcomes ranging from transient deficits to irreversible paralysis. The clinical manifestations vary depending on the level and severity of the injury and may include motor and sensory deficits, bowel and bladder dysfunction, chronic pain, and autonomic disturbances. Furthermore, patients with SCI are susceptible to secondary complications such as respiratory compromise, circulatory instability, and psychological distress, which significantly impact long-term outcomes and quality of life.^[7,8]^

Systematic reviews have highlighted key early predictors of functional outcomes after traumatic SCI, including initial neurological status, injury level, and age, with incomplete injuries showing better recovery potential.^[9,10]^ Long-term studies emphasize the role of rehabilitation in achieving ambulatory status and return to work, yet gaps persist in understanding fracture morphology’s impact on prognosis.^[11,12]^ While these reviews provide global insights, data from high-volume trauma centers in developing regions like Pakistan remain limited, particularly regarding fracture-specific recovery patterns and post-discharge neurological improvements. This study addresses this gap by quantifying associations in a prospective cohort from such a setting.

### 1.1. Objective

To assess the clinical characteristics, fracture patterns, and neurological outcomes of spinal injuries and to identify factors associated with functional recovery through bivariate analyses.

### 1.2. Material and methods

This was a prospective cohort study conducted over 24 months, from January 2023 to December 2024, at the Department of Neurosurgery, Lady Reading Hospital, Peshawar. As a high-volume tertiary care center, the hospital caters to spinal trauma patients from across Khyber Pakhtunkhwa and surrounding regions. The study was approved by the institutional ethical review board, and written informed consent was obtained from all participants before enrollment.

Patients were enrolled consecutively using a non-probability sampling technique. Eligible participants included adults aged 18 years or older of either gender who sustained traumatic spinal injuries as a result of RTAs, diving into shallow water, or falls from height (height of fall). Patients with non-traumatic spinal pathology, pathological fractures, or incomplete follow-up data were excluded. An additional 27 patients were excluded due to incomplete data or loss to follow-up. A total of 123 patients met the inclusion criteria and were enrolled in the study. Patients were followed longitudinally to assess outcomes at admission, discharge, and three-month follow-up.

Data were collected prospectively using a structured proforma at the time of admission, during hospital stay, and at three-month follow-up. Demographic and clinical variables included age, gender, mechanism of injury, Glasgow Coma Scale score at presentation, time from injury to hospital arrival, use of spinal immobilization during transport, and presence of associated injuries. Each patient underwent a detailed neurological examination and imaging. The level and type of spinal fracture were documented, and fracture classification was performed using the Arbeitsgemeinschaft für Osteosynthesefragen Spine classification system. Neurological status was assessed using the American Spinal Injury Association (ASIA) Impairment Scale at 3 time points: on admission, at hospital discharge, and the three-month follow-up.

Management approach, either surgical or conservative, was recorded. For patients undergoing surgery, details of the surgical approach (anterior, posterior, or combined) and specific procedures, such as anterior cervical discectomy and fusion (ACDF), anterior cervical corpectomy and fusion, or both, were documented. Additional clinical parameters, including the number of days spent in the intensive care unit, in-hospital complications, physiotherapy sessions received, and total hospital stay, were also recorded. Functional outcomes were assessed using ASIA grade and the visual analogue scale (VAS) for pain at the three-month follow-up.

All data were analyzed using IBM SPSS Statistics version 25. Descriptive statistics were used to summarize patient demographics and clinical characteristics. Categorical variables were expressed as frequencies and percentages, while continuous variables were reported as means and standard deviations. Associations between clinical variables – such as mechanism of injury, fracture type, and intervention type – and outcomes were examined using chi-square tests. Effect sizes were reported as percentage-point differences and odds ratios, where applicable. Due to sample size limitations, multivariable modeling was not performed; bivariate associations are reported. Longitudinal changes in ASIA grades across the 3 time points (admission, discharge, follow-up) were evaluated using the Friedman test. Post hoc pairwise comparisons were conducted using the Wilcoxon signed-rank test to assess the significance of neurological improvement over time. A *P*-value of <.05 was considered statistically significant.

Efforts were made to minimize bias by using standardized assessment tools and protocols. Trained neurosurgical residents conducted neurological evaluations to ensure consistency. Measurement bias was further reduced by applying uniform imaging protocols and objective classification systems for fractures and neurological grading. The study employed complete-case analysis, excluding patients with missing or incomplete follow-up data from the final analysis. The study size was determined by the number of eligible patients who presented during the defined study period, as the research aimed to describe patterns and associations rather than to test a specific hypothesis through sample size calculation. Quantitative variables, such as age and VAS scores, were treated as continuous, while categorical variables, such as ASIA grades and fracture types, were analyzed according to clinically relevant groupings.

## 2. Results

### 2.1. Clinical characteristics

The study included 123 patients with spinal injuries. The mean age was 32.2 ± 11.4 years. The average Glasgow Coma Scale score at presentation was 11.3 ± 6.5. The mean duration of intensive care unit stay was 0.36 ± 1.80 days, and the mean total hospital stay was 8.4 ± 4.3 days.

Of the total cohort, 81 (65.9%) were male and 42 (34.1%) were female. The most frequent mechanism of injury was RTAs, accounting for 61 (49.6%) cases, followed by falls in 43 (35.0%) and diving injuries in 19 (15.4%) patients. The time to hospital presentation was within 6 hours for 18 (14.6%) patients, 6 to 12 hours for 28 (22.8%), 12 to 24 hours for 48 (39.0%), and more than 24 hours in 29 (23.6%) patients.

Spinal immobilization during transport was reported in 99 (80.5%) patients, while 24 (19.5%) were not immobilized. Associated injuries included limb injuries in 33 (26.8%) patients, thoracic injuries in 23 (18.7%), head injuries in 19 (15.4%), abdominal injuries in 5 (4.1%), pelvic injuries in 5 (4.1%), and other injuries in 13 (10.6%). No associated injury was present in 25 (20.3%) patients. On admission, ASIA grading revealed 52 (42.3%) patients with Grade A, 8 (6.5%) with Grade B, 26 (21.1%) with Grade C, 25 (20.3%) with Grade D, and 12 (9.8%) with Grade E (Table 1).

**Table 1:** Patients’ arrival characteristics and clinical variables (n = 123).

Variable	Categories	n (%)
Gender	Male	81 (65.9)
	Female	42 (34.9)
Mechanism of injury	RTA	61 (49.6)
	Diving	19 (15.4)
	Fall	43 (35.0)
Time to hospital presentation	<6 h	18 (14.6)
	6–12 h	28 (22.8)
	12–24 h	48 (39.0)
	>24 h	29 (23.6)
Spinal immobilization	Yes	99 (80.5)
	No	24 (19.5)
Associated injuries	Abdominal	5 (4.1)
	Head	19 (15.4)
	Limb	33 (26.8)
	None	25 (20.3)
	Others	13 (10.6)
	Pelvic	5 (4.1)
	Thoracic	23 (18.7)
ASIA grade on admission	Grade A	52 (42.3)
	Grade B	8 (6.5)
	Grade C	26 (21.1)
	Grade D	25 (20.3)
	Grade E	12 (9.8)

ASIA = American Spinal Injury Association, RTA = road traffic accident.

## 3. Common patterns of vertebral fracture

The most commonly involved vertebral levels were L1 to L2 in 30 (24.4%) patients, and cervical spine levels (C1–C6, including spinal cord injury without radiologic abnormality [SCIWORA]) also in 30 (24.4%) patients. Other affected regions included D9 to D12 in 25 (20.3%), L3 to L5 in 14 (11.4%), D5 to D8 in 12 (9.8%), and D1 to D4 in 7 (5.7%) patients.

The most common fracture type was burst fracture, observed in 70 (56.9%) patients, followed by wedge fractures in 31 (25.2%), facet dislocations in 7 (5.7%), tear drop fractures in 6 (4.9%), SCIWORA in 5 (4.1%), and ligamentous injuries in 4 (3.3%) (Table 2).

**Table 2:** Pattern of fractures.

Variable	Categories	Frequency (%)
Fracture level	L1–L2	30 (24.4)
	L3–L5	14 (11.4)
	D1–D4	7 (5.7)
	D5–D8	12 (9.8)
	D9–D12	25 (20.3)
	Cervical (C1–C6) incl. SCIWORA	30 (24.4)
Fracture type	Burst	70 (56.9)
	Wedge	31 (25.2)
	Ligamentous	4 (3.3)
	SCIWORA	5 (4.1)
	Facet dislocation	7 (5.7)
	Tear drop	6 (4.9)

SCIWORA = spinal cord injury without radiological abnormality.

## 4. Surgical and conservative management

Surgical intervention was undertaken in 83 (67.5%) patients, while 40 (32.5%) were managed conservatively. Among those operated upon, a posterior surgical approach was utilized in 60 (48.8%) patients, an anterior approach in 17 (13.8%), and a combined anterior-posterior approach in 6 (4.9%).

With regard to surgical procedures, posterior fusion was the most common, performed in 59 (48.8%) patients. Anterior cervical corpectomy and fusion was carried out in 13 (10.7%), ACDF in 4 (3.3%), and ACDF combined with posterior fusion in 4 (3.3%) patients. A total of 41 (33.9%) patients were managed non-operatively (Table 3).

**Table 3:** Surgical statistics.

Variable	Groups	Frequency (%)
Primary intervention	Surgical	83 (67.5)
	Conservative	40 (32.5)
Surgical approach	Anterior	17 (13.8)
	Posterior	60 (48.8)
	Anterior + Posterior	6 (4.9)
	Conservative	40 (32.5)
Procedure type	ACCF	13 (10.7)
	ACDF	4 (3.3)
	ACDF + Posterior	4 (3.3)
	Posterior fusion	59 (48.8)
	Conservative	41 (33.9)

ACCF = anterior cervical corpectomy and fusion, ACDF = anterior cervical discectomy and fusion.

## 5. In-hospital complications and discharge outcomes

In-hospital complications were recorded in 21 (17.1%) patients. These included chest infections in 11 (8.9%), cerebrospinal fluid leaks in 6 (4.9%), and deep vein thrombosis in 4 (3.3%). The remaining 102 (82.9%) patients had an uneventful hospital stay.

ASIA grade at discharge was Grade A in 49 (39.8%) patients, Grade B in 7 (5.7%), Grade C in 20 (16.3%), Grade D in 35 (28.5%), and Grade E in 12 (9.8%).

## 6. Follow-up outcomes

At follow-up, 76 (61.8%) patients were ambulatory, 25 (20.3%) were wheelchair-bound, and 22 (17.9%) remained bedridden. ASIA grading at follow-up showed 38 (30.9%) patients remained at Grade A, 8 (6.5%) at Grade B, 10 (8.1%) at Grade C, 22 (17.9%) at Grade D, and 45 (36.6%) had improved to Grade E. Physiotherapy was received by 111 (90.2%) patients.

Pain assessment using the VAS revealed mild pain in 76 (61.8%) patients, moderate pain in 39 (31.7%), and severe pain in 8 (6.5%) (Table 4).

**Table 4:** Post-operative descriptive statistics.

Variable	Categories	Frequency (%)
In-hospital complications	None	102 (82.9)
	CSF leak	6 (4.9)
	DVT	4 (3.3)
	Chest infection	11 (8.9)
ASIA grade at discharge	Grade A	49 (39.8)
	Grade B	7 (5.7)
	Grade C	20 (16.3)
	Grade D	35 (28.5)
	Grade E	12 (9.8)
ASIA grade at follow-up	Grade A	38 (30.9)
	Grade B	8 (6.5)
	Grade C	10 (8.1)
	Grade D	22 (17.9)
	Grade E	45 (36.6)
Physiotherapy received	Yes	111 (90.2)
	No	12 (9.8)
Ambulatory status at follow-up	Ambulatory	76 (61.8)
	Wheelchair-bound	25 (20.3)
	Bedridden	22 (17.9)
VAS score at follow-up	Mild (1–3)	76 (61.8)
	Moderate (4–6)	39 (31.7)
	Severe (7–10)	8 (6.5)

ASIA = American Spinal Injury Association, CSF = cerebrospinal fluid, DVT = deep venous thrombosis, VAS = visual analogue scale.

## 7. Neurological outcome trajectory

**Figure 1.**
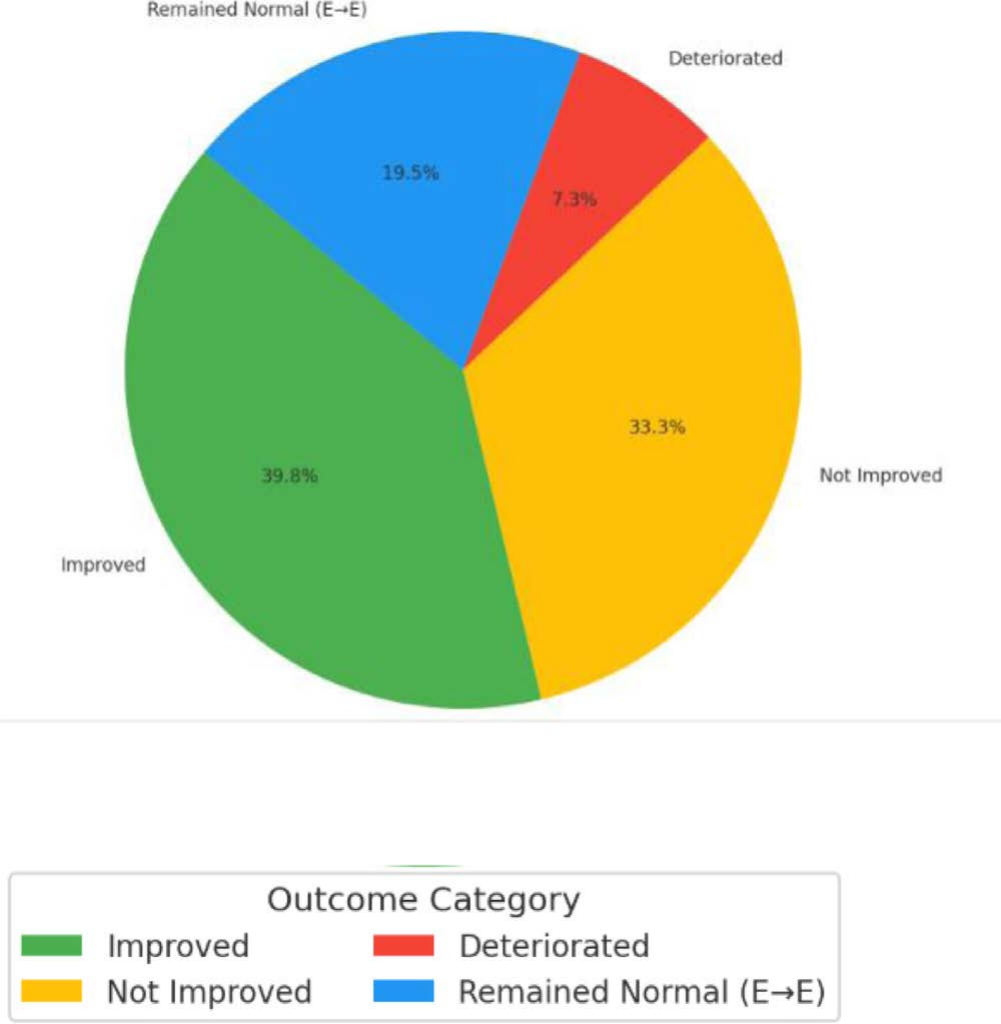
Neurological outcomes on follow-up after management by ASIA grading. ASIA = American Spinal Injury Association.

Neurological improvement was observed in 49 (39.8%) patients, while 41 (33.3%) showed no change. A total of 9 (7.3%) experienced deterioration, and 24 (19.5%) maintained normal neurological status (Fig. 1).

## 8. Association between fracture type and neurological outcome

Fracture type distribution was analyzed across various clinical parameters in Table 5. No significant difference was found regarding the mechanism of injury (*P* = .103). However, ambulatory status and ASIA grade at follow-up were significantly associated with fracture type (*P* < .001), with wedge fractures and SCIWORA demonstrating better outcomes (e.g., 96.8% ambulatory for wedge vs 41.4% for burst, 55.4 percentage point difference). Pain severity also varied substantially by fracture type (*P* = .001) (e.g., 93.5% mild pain for wedge vs 50.0% for burst, 43.5 percentage point difference). In contrast, in-hospital complication rates did not differ significantly by fracture type (*P* = .174). Surgical treatment was more frequently applied in patients with burst fractures, facet dislocations, and teardrop fractures (*P* < .001).

**Table 5:** Association of fracture type with various clinical variables (n = 123).

Variable	Categories	Burst n (%)	Wedge n (%)	Ligamentous n (%)	SCIWORA n (%)	Facet dislocation n (%)	Tear drop n (%)	*P*-value
Mechanism of injury	RTA	33 (46.5)	19 (61.3)	2 (50.0)	3 (60.0)	3 (42.9)	1 (16.7)	.103
	Diving	16 (22.5)	1 (3.2)	0 (0.0)	0 (0.0)	2 (28.6)	0 (0.0)	
	Fall	21 (30.0)	11 (35.5)	2 (50.0)	2 (40.0)	2 (28.6)	5 (83.3)	
Follow-up outcome	Ambulatory	29 (41.4)	30 (96.8)	3 (75.0)	5 (100.0)	4 (57.1)	5 (83.3)	<.001
	Bedridden	19 (27.1)	0 (0.0)	1 (25.0)	0 (0.0)	2 (28.6)	0 (0.0)	
	Wheelchair-bound	22 (31.4)	1 (3.2)	0 (0.0)	0 (0.0)	1 (14.3)	1 (16.7)	
ASIA grade follow-up	A	33 (47.1)	1 (3.2)	1 (25.0)	0 (0.0)	2 (28.6)	1 (16.7)	<.001
	B	7 (10.0)	0 (0.0)	0 (0.0)	0 (0.0)	1 (14.3)	0 (0.0)	
	C	6 (8.6)	1 (3.2)	0 (0.0)	1 (20.0)	0 (0.0)	2 (33.3)	
	D	15 (21.4)	0 (0.0)	2 (50.0)	2 (40.0)	1 (14.3)	2 (33.3)	
	E	9 (12.9)	29 (93.5)	1 (25.0)	2 (40.0)	3 (42.9)	1 (16.7)	
VAS at follow-up	Mild	35 (50.0)	29 (93.5)	3 (75.0)	3 (60.0)	4 (57.1)	2 (33.3)	.001
	Moderate	30 (42.9)	2 (6.5)	0 (0.0)	2 (40.0)	3 (42.9)	2 (33.3)	
	Severe	5 (7.1)	0 (0.0)	1 (25.0)	0 (0.0)	0 (0.0)	2 (33.3)	
In-hospital complications	None	50 (71.4)	31 (100.0)	4 (100.0)	5 (100.0)	6 (85.7)	6 (100.0)	.174
	CSF leak	6 (8.6)	0 (0.0)	0 (0.0)	0 (0.0)	0 (0.0)	0 (0.0)	
	DVT	3 (4.3)	0 (0.0)	0 (0.0)	0 (0.0)	1 (14.3)	0 (0.0)	
	Chest infection	11 (15.7)	0 (0.0)	0 (0.0)	0 (0.0)	0 (0.0)	0 (0.0)	
Primary intervention	Surgical	67 (95.7)	3 (9.7)	0 (0.0)	0 (0.0)	7 (100.0)	6 (100.0)	<.001
	Conservative	3 (4.3)	28 (90.3)	4 (100.0)	5 (100.0)	0 (0.0)	0 (0.0)	

χ^2^ or Fisher exact test was used to determine the significance.

*P* -value of ≤.05 was considered significant.

ASIA = American Spinal Injury Association, CSF = cerebrospinal fluid, DVT = deep venous thrombosis, SCIWORA = spinal cord injury without radiologic abnormality, RTA = road traffic accident, VAS = visual analogue scale.

## 9. Predictors of neurological improvement

In order to check the improvement status, we excluded those patients who presented with ASIA grade E on arrival and remained grade E on follow-ups to remove bias. In Table 5, neurological improvement was significantly associated with fracture type (*P* < .001), primary intervention (*P* < .001), higher ASIA grade on admission (*P* < .001), physiotherapy (*P* = .047), and in-hospital complications (*P* = .047). No significant association was observed for injury mechanism, time to hospital, or spine immobilization. Table 6. For initial ASIA grade, effect sizes showed a 73.1 percentage point difference in improvement rates between grade D (100%) and A (26.9%), with approximate odds ratio 15.2 (95% CI: 5.8–39.9).

**Table 6:** Association of variables with neurological improvement status (n = 111).

Variable	Categories	Improved n (%)	Not improved n (%)	*P*-value
Mechanism of injury	RTA	32 (48.5)	25 (55.6%)	.396
	Diving	7 (10.6)	7 (15.6%)	
	Fall	27 (40.9)	13 (28.9%)	
Time to hospital presentation	<6 h	8 (12.1)	8 (17.8%)	.860
	6–12 h	14 (21.2)	9 (20.0%)	
	12–24 h	28 (42.4)	17 (37.8%)	
	>24 h	16 (24.2)	11 (24.4%)	
Spine immobilised	Yes	49 (74.2)	39 (86.7%)	.113
	No	17 (25.8)	6 (13.3%)	
Fracture type	Burst	25 (37.9)	39 (86.7%)	<.001
	Wedge	24 (36.4)	1 (2.2%)	
	Ligamentous	3 (4.5)	1 (2.2%)	
	SCIWORA	5 (7.6)	0 (0.0%)	
	Facet dislocation	5 (7.6)	2 (4.4%)	
	Tear drop	4 (6.1)	2 (4.4%)	
Primary intervention	Surgical	36 (54.5)	44 (97.8%)	<.001
	Conservative	30 (45.5)	1 (2.2%)	
ASIA grade on admission	Grade A	14 (21.2)	38 (84.4%)	<.001
	Grade B	4 (6.1)	4 (8.9%)	
	Grade C	23 (34.8)	3 (6.7%)	
	Grade D	25 (37.9)	0 (0.0%)	
Physiotherapy received	Yes	63 (95.5)	38 (84.4%)	.047
In-hospital complications	None	59 (89.4)	31 (68.9%)	.047
	CSF leak	2 (3.0)	4 (8.9%)	
	DVT	2 (3.0)	2 (4.4%)	
	Chest infection	3 (4.5)	8 (17.8%)	

χ^2^ or Fisher exact test was used to determine significance.

A *P*-value of ≤.05 was considered significant.

ASIA = American Spinal Injury Association, CSF = cerebrospinal fluid, DVT = deep venous thrombosis, SCIWORA = spinal cord injury without radiologic abnormality, RTA = road traffic accident.

## 10. Longitudinal ASIA grade comparison

ASIA grades were compared across 3 time points: admission, discharge, and three-month follow-up using the Friedman test. A statistically significant improvement was observed (χ^2^ = 125.05, df = 2, *P* < .001), indicating neurological recovery over time. Pairwise Wilcoxon signed-rank tests revealed that 13% (n = 16) of patients improved by discharge, while 56% (n = 69) showed neurological improvement by follow-up compared to baseline. Additionally, 53% (n = 65) continued to improve between discharge and follow-up. No patient showed deterioration during the follow-up period. These findings suggest that a significant portion of neurological recovery occurred after hospital discharge, underscoring the potential benefit of continued rehabilitation and follow-up care Table 7.

**Table 7:** Comparison of ASIA grades over time (n = 123).

Comparison	Improved	No change	Deteriorated	Mean rank	*P*-value
Admission → Discharge	16 (13.0%)	106 (86.2%)	1 (0.8%)	1.80	–
Admission → Follow-up	69 (56.1%)	54 (43.9%)	0 (0.0%)	2.54	–
Discharge → Follow-up	65 (52.8%)	58 (47.2%)	0 (0.0%)	–	–
Overall (Friedman test)	–	–	–	–	**<.001**

Friedman test χ² = 125.055, df = 2, *P* < .001.

The Wilcoxon signed-rank test is used for pairwise comparisons.

ASIA = American Spinal Injury Association.

## 11. Discussion

This study provides novel quantitative insights into differential neurological recovery patterns across fracture types in SCI, revealing clinically significant disparities beyond previous qualitative observations. Our findings contribute key advances: quantified recovery probabilities by fracture morphology (e.g., 8-fold difference in normal function between wedge and burst fractures), evidence for substantial post-discharge improvement (53% continued gains), and refined prognostic indicators for decision-making in resource-limited settings. Our cohort had a male predominance (65.9%) with a mean age of 32.2 ± 11.4 years, which aligns with previous studies showing that SCI predominantly affect young males in their most productive years. Faleiros F et al and Toluse AM et al similarly reported a male-to-female ratio of approximately 2:1 in their extensive cohort study, aligning with our demographic findings.^[13,14]^

RTAs were the leading cause of injury (49.6%), followed by falls (35.0%) and diving injuries (15.4%) in our study. This pattern aligns with the findings of Barbiellini Amidei C et al and Miyakoshi N et al, who also identified motor vehicle accidents as the predominant mechanism in developing countries.^[15,16]^ Conversely, Zhang ZR et al, Mittal S et al, and Yokogawa N et al reported that falls are the leading cause of injury in their elderly populations, highlighting the importance of age-specific injury.^[6,17,18]^

The most frequently affected spinal levels were L1 to L2 (24.4%) and cervical levels (24.4%), with burst fractures representing the most common fracture type (56.9%) in our study. These findings are supported by Cahueque M et al, who reported similar thoracolumbar junction involvement in their series.^[19]^ Our quantified 80.6 percentage-point difference in normal function rates between wedge (93.5%) and burst (12.9%) fractures surpasses the variations reported in prior studies,^[20]^ providing concrete data for prognostication in similar populations.

At follow-up, 61.8% of patients achieved ambulatory status, with significant improvement in ASIA grades from admission to follow-up. Similar findings are reported by Mahmoodkhani M et al.^[16]^ The proportion of patients with complete injuries (ASIA Grade A) decreased from 42.3% at admission to 30.9% at follow-up, indicating meaningful neurological recovery in our series. In contrast, DiPiro ND et al achieved superior functional outcomes with an 84% ambulatory rate, which might be due to differences in rehabilitation protocols or follow-up duration.^[21]^ The 55.4 percentage point difference in ambulatory rates by fracture type highlights the prognostic value of morphology, aligning with systematic reviews on early predictors.^[9,10]^

Our analysis showed that neurological improvement was significantly associated with ASIA grading, with a higher ASIA grade at admission associated with better improvement, as reported by Wilson JR et al and Stenimahitis V et al.^[22,23]^ It was also significantly associated with physiotherapy (*P* = .047), suggesting that those who underwent physiotherapy had better neurological outcomes. It is supported by the findings of Mazwi et al.^[24]^ The 41.2 percentage-point difference in improvement with physiotherapy underscores its role in extended recovery, with implications for outpatient programs.^[11,12]^

Compared to wedge fracture, burst fracture in our series had poor outcomes neurologically, which aligns with the study findings of Goulet J et al.^[25]^ SCIWORA showed the most favorable outcomes, which aligns with Qi C et al’s^[26]^ findings regarding the prognostic significance of fracture morphology. Adegeest CY et al^[27]^ and Essa A et al^[28]^ found an association between surgical timing and neurological recovery, aligning with our findings that surgical intervention was associated with improved outcomes. However, conservative management showed higher improvement rates (96.8% vs 45.0%, 51.8 percentage point difference), likely due to the selection of stable cases and refining the criteria for surgical selection.

This study has several limitations. The single-center nature and modest sample size limit generalizability and preclude robust multivariable modeling due to quasi-separation in categories (e.g., 100% improvement in ASIA D). The three-month follow-up is short for long-term outcomes, and rehabilitation protocols were not standardized. Future multicenter studies with extended follow-up, standardized interventions, and adjusted analyses would strengthen these findings.

## 12. Conclusions

This study demonstrates that fracture type, treatment modality, and rehabilitation significantly influence neurological outcomes in spinal injuries. The superior outcomes observed in wedge fractures and SCIWORA, combined with the positive impact of surgical intervention and physiotherapy, are notable. These quantified associations provide clinicians with prognostic tools for patient counseling, tailored rehabilitation, and early identification of high-risk cases, thereby improving long-term functional recovery and quality of life.

## Author contributions

**Conceptualization:** Zia Ur Rehman.

**Data curation:** Zia Ur Rehman.

**Formal analysis:** Muhammad Sohaib Khan.

**Software:** Muhammad Sohaib Khan.

**Supervision:** Bilal Khan.

**Writing – original draft:** Syed Shayan Shah.

**Writing – review & editing:** Muhammad Sohaib Khan, Bilal Khan, Syed Jawad Ahmad, Muhammad Aamir, Zia Ur Rehman.
